# Sociotechnical Cross-Country Analysis of Contextual Factors That Impact Patients’ Access to Electronic Health Records in 4 European Countries: Framework Evaluation Study

**DOI:** 10.2196/55752

**Published:** 2024-08-26

**Authors:** Jonas Moll, Isabella Scandurra, Annika Bärkås, Charlotte Blease, Maria Hägglund, Iiris Hörhammer, Bridget Kane, Eli Kristiansen, Peeter Ross, Rose-Mharie Åhlfeldt, Gunnar O Klein

**Affiliations:** 1 Centre for Empirical Research on Information systems School of Business Örebro University Örebro Sweden; 2 Participatory eHealth and Health Data Research Group Department of Women's and Children's Health Uppsala University Uppsala Sweden; 3 Medtech Science & Innovation Centre Uppsala University Hospital Uppsala Sweden; 4 Digital Psychiatry Department of Psychiatry Beth Israel Deaconess Medical Center Harvard Medical School Boston, MA United States; 5 Department of Computer Science Aalto University Espoo Finland; 6 Karlstad University Business School Karlstad Sweden; 7 Norwegian Centre for E-health Research University Hospital of North Norway Tromsø Norway; 8 E-Medicine Centre Department of Health Technologies Tallinn University of Technology Tallinn Estonia; 9 Research Department East Tallinn Central Hospital Tallinn Estonia; 10 School of Informatics University of Skövde Skövde Sweden

**Keywords:** electronic health record, EHR, health data, national survey, web-based medical record, web-based record access, patient access, patient-accessible electronic health record, patient portal, sociotechnical analysis

## Abstract

**Background:**

The NORDeHEALTH project studies patient-accessible electronic health records (PAEHRs) in Estonia, Finland, Norway, and Sweden. Such country comparisons require an analysis of the sociotechnical context of these services. Although sociotechnical analyses of PAEHR services have been carried out in the past, a framework specifically tailored to in-depth cross-country analysis has not been developed.

**Objective:**

This study aims to develop and evaluate a method for a sociotechnical analysis of PAEHRs that advances a framework for sociotechnical analysis of eHealth solutions first presented by Sittig and Singh. This first article in a series presents the development of the method and a cross-country comparison of the contextual factors that enable PAEHR access and use.

**Methods:**

The dimensions of the framework for sociotechnical analysis were thoroughly discussed and extended in a series of workshops with international stakeholders, all being eHealth researchers focusing on PAEHRs. All countries were represented in the working group to make sure that important national perspectives were covered. A spreadsheet with relevant questions related to the studied services and the various dimensions of the sociotechnical framework was constructed and distributed to the 4 participating countries, and the project participants researched various national sources to provide the relevant data for the comparisons in the 10 sociotechnical dimensions.

**Results:**

In total, 3 dimensions were added to the methodology of Sittig and Singh to separate clinical content from features and functions of PAEHRs and demonstrate basic characteristics of the different countries regarding national and regional steering of health care and information and communications technology developments. The final framework contained the following dimensions: metadata; hardware and software computing infrastructure; features and functions; clinical content shared with patients; human-computer interface; people; workflow and communication; the health care organization’s internal policies, procedures, and culture; national rules, regulations, and incentives; system measurement and monitoring; and health care system context. The dimensions added during the study mostly concerned background information needed for cross-country comparisons in particular. Several similarities were identified among the compared countries, especially regarding hardware and software computing infrastructure. All countries had, for example, one national access point, and patients are provided a PAEHR automatically. Most of the differences could be identified in the *health care system context* dimension. One important difference concerned the governing of information and communications technology development, where different levels (state, region, and municipality) were responsible in different countries.

**Conclusions:**

This is the first large-scale international sociotechnical analysis of services for patients to access their electronic health records; this study compared services in Estonia, Finland, Norway, and Sweden. A methodology for such an analysis was developed and is presented to enable comparison studies in other national contexts to enable future implementations and evaluations of PAEHRs.

## Introduction

### Background

Patients’ web-based access to their electronic health records (EHRs) is increasingly being implemented internationally [1-3], and a growing body of literature indicates strong benefits to patients, including understanding their care plans better [4], feeling more in control of their care [4,5], being better informed about their medication [6], improved communication with and trust in their clinicians [5,7], and improved patient safety [8].

Patient-accessible EHRs (PAEHRs) are web-based services providing patients with secure access to view and sometimes edit or comment on their EHRs made available by their health care providers [9]. A PAEHR is directly linked to the EHRs, which are shared patient records entered and maintained by health care service providers and contain historical data about a patient [10]. Medical and health data and information in the EHR are created and managed by authorized providers in a digital format capable of being shared with other providers across more than one health care organization [11]. In addition, a PAEHR may include access to the services supporting a person’s access to health care services (eg, e-booking and e-consultation) and to evidence-based tools (eg, patient guidelines, educational materials, and reimbursement information). It is important to distinguish between PAEHRs and personal health records—the latter being a health record that the patients themselves control and maintain to track their own health, for example, on paper; in Microsoft Excel sheets; or, predominantly, in health care apps. Personal health records are external applications that can potentially be linked with EHRs to enable sharing [10].

In this study, we focused on national services in the 4 countries of Estonia, Finland, Norway, and Sweden and excluded web-based services designed to provide access to the services of only 1 health care provider. Among the studied countries, Estonia was the first to offer citizens web-based access to their EHR through the service Digilugu that was launched nationwide in 2008 [12]. The Finnish counterpart, Omakanta, was launched nationwide in 2010 with only limited functionality and reached full functionality in 2015 after a step-by-step adoption of functions [13]. In Sweden, the national PAEHR service Journalen was launched in the Region Uppsala in 2012 and has been accessible to all citizens since 2018 [14]. In Norway, the national PAEHR service Helsenorge was first launched in 1 of 4 regions in 2015, and as of 2023, citizens in 3 out of 4 regions can use the service to access their PAEHRs [15].

Despite the reported benefits of PAEHRs and patients’ web-based record access, implementation is often slow and challenging, and the complexity of health care systems and technical infrastructure leads to great diversity in, for example, the information to which patients are given access and when they can access it across regions and health care settings. Similarly, patients’ adoption and use of PAEHRs also varies across contexts. To understand why these differences exist and better adapt the design and implementation of PAEHRs to a specific context, there is a need for a more fine-grained understanding of the social and technical underpinnings of this innovation.

### Sociotechnical Systems

As described by Baxter and Sommerville [16], the problems that arise when designing and implementing complex IT systems are not just technical, engineering problems. These systems are developed and operated by people working in organizations that inevitably have different, often conflicting goals and views on the role and design of the system. The IT system is part of a broader “sociotechnical” system, and to understand success factors and barriers to implementing web-based record access and identify best practices and guidelines, there is a need to approach these eHealth services as complex sociotechnical systems.

Sittig and Singh [17] have proposed a multidimensional sociotechnical framework in which any health IT innovation, intervention, application, or device implemented within a complex adaptive health care system can be studied. The sociotechnical framework by Sittig and Singh [17] identifies eight dimensions of sociotechnical systems in health care that need to be considered in both development and evaluation: (1) hardware and software computing infrastructure; (2) clinical content; (3) human-computer interface; (4) people; (5) workflow and communication; (6) internal organizational policies, procedures, and culture; (7) external rules, regulations, and pressures; and (8) system measurement and monitoring [17]. The framework breaks down components of the technology to enable researchers to identify specific problems with implementation. It also includes monitoring processes and government structures that need to be in place for the system to achieve its goals. The interrelatedness of the components makes the framework pertinent when eHealth technologies and users are at the core of the investigation.

In 2017, Hägglund and Scandurra [18] began analyzing the Swedish PAEHR system from a sociotechnical perspective using the framework by Sittig and Singh [17], and their results laid the foundation for continued work within the NORDeHEALTH research project [19] involving partners from Estonia, Finland, Norway, and Sweden. These countries were deemed appropriate for the development and first test of the cross-country sociotechnical analysis method derived in this study as they have comparable government and health care structures. In addition, they have all reached maturity in the use of their respective PAEHR systems—these countries are all among the early adopters of this specific type of eHealth service for patients. In a 2021 workshop, a first draft of an extended version of the framework by Sittig and Singh [17] as a sociotechnical template for analyzing PAEHR implementations was introduced and discussed [20]. However, the original framework by Sittig and Singh [17] needed to be more specifically adapted to PAEHRs and their contexts to enable useful cross-country comparisons, a process that will be described further in this paper.

The full results of the sociotechnical analysis of the 4 countries involved in the NORDeHEALTH project are being published in an article series; it is intended for each publication to offer results related to specific and related framework dimensions and, thereby, focus on different themes. This paper is intended to offer a detailed description of the overarching research method as well as a sociotechnical analysis of dimensions providing the context for other articles of the series.

### Aims and Research Questions

The aims of this study were to (1) develop a detailed collaborative method suitable for cross-country sociotechnical analyses of PAEHRs and (2) use the data collection to compare contextual factors that enable PAEHR access and use.

Although the proposed work process, including the production of an extensive sociotechnical analysis template, is described in the context of comparing PAEHRs in 4 specific countries, it can be adjusted to cover other eHealth services for patients across other nations. The comparison and discussion regarding the national contexts will provide a robust foundation and understanding of other parts of the sociotechnical analysis, such as included features and national and regional incentives for the use and promotion of PAEHRs. The following research questions (RQs) guided the work presented in this paper:

- RQ 1: how can a sociotechnical framework for health IT be adapted and a collaborative method be developed that is suitable for cross-country sociotechnical analyses of PAEHR services?
- RQ 2: how can the method be used to compare the PAEHRs in Estonia, Finland, Norway, and Sweden?

## Methods

### Research Method

This study has 2 main parts inspired by different phases in the design science methodology [21]. In the first part, a collaborative method, including a data collection template, for sociotechnical cross-country analysis of PAEHRs was derived: (1) problem identification and motivation; that is, based on earlier experiences with the framework by Sittig and Singh [17] and the fact that the framework is not adapted for comparisons across contexts, the following problem was defined, how can we conduct fruitful comparative analysis among PAEHRs in different regions and health care settings? (2) objectives of a solution; that is, due to the identified need to perform a complete sociotechnical comparison among health care contexts, the main objective of the new artifact (being a method for collecting data, including a refined framework and a data collection instrument) is to enable a comparison that would consider the sociotechnical contexts of PAEHRs; and (3) design and development; that is, a dedicated collaborative method was developed with a focus on adjusting the framework for cross-country comparisons and deriving a data collection template enabling the collection of all relevant data from the specific countries involved.

The second part of our study (in this paper referred to as *data collection*) consists of the collection and comparison of the sociotechnical characteristics of the 4 countries participating in the NORDeHEALTH project: Estonia, Finland, Norway, and Sweden. This can be seen as case studies of the more general questions regarding the availability and use of the PAEHR in a country. However, it is also an evaluation of the method as an artifact in the design science paradigm: (1) demonstration—the data collection template derived from the first part of the study was used to conduct a cross-country comparison among 4 Nordic countries—and (2) evaluation—finally, the results from the comparison were analyzed to evaluate whether the approach provides an understanding that explains differences among countries in the adoption of PAEHRs by regions and providers and patients.

In the remaining sections, the different points of the process are elaborated on, with emphasis on the design and development, demonstration, and evaluation steps. The work was led by a core analysis team (JM, IS, GK, and AB) of health informatics researchers.

### Development of the Collaborative Method

The development of the data collection instrument based on the sociotechnical framework proposed by Sittig and Singh [17] started before the NORDeHEALTH project had begun.

In 2017, a first sociotechnical analysis of the Swedish PAEHR service Journalen was conducted with the aim of increasing the understanding of factors that influence the design, implementation, adoption, and use of the service [18]. However, the analysis did not go into the details of each dimension of the framework but, rather, highlighted some overall challenges. The results of this early analysis were used as input to an international workshop held in 2021, for which a more detailed template specifically adapted to PAEHRs was developed (Textbox 1). In addition to including specific PAEHR-related questions to each dimension, a new dimension, *Features and functions*, was added. Some other dimensions were renamed. During the workshop, international experts provided feedback on the template that was incorporated into further refinements, leading to the data collection form presented in this study.

**The sociotechnical framework dimensions included in an early version of the data collection template following the work by Sittig and Singh.**

**Dimension and description**

- Hardware and software computing infrastructure: focuses only on the hardware and software required to run the applications.
- Features and functions: important features and functions in the patient-accessible electronic health record (PAEHR) service or in related services. As there is not yet a strict definition of what functionality is included in a PAEHR or not, we included functions that may be considered external to a PAEHR in some contexts.
- Clinical content shared with patients: includes everything on the data-information-knowledge continuum that is stored in the PAEHR service and made accessible to patients.
- Human-computer interface: focuses on the usability of the PAEHR service.
- People: represents the humans involved in all aspects of the implementation and use of the eHealth application and how they experience the use.
- Workflow and communication: focuses on collaboration and communication among different users and assessing how well the eHealth application supports the current clinical workflow.
- The health care organization’s internal policies, procedures, and culture: affects every other dimension in this model as it includes any internal IT policy documents and managerial procedures that may influence the implementation and use of eHealth.
- National rules, regulations, and incentives: focuses on external forces that facilitate or place constraints on the design, development, implementation, use, and evaluation of eHealth in the respective clinical settings.
- System measurement and monitoring: focuses on the need for an effective system measurement and monitoring program to identify the availability of features and functions and how they are used as well as expected outcomes and unintended consequences of the PAEHR service.

The resulting Microsoft Excel template was then used as a basis for developing a complete data collection form within the scope of the NORDeHEALTH project, including questions, response options, and comment sections for all the dimensions in the sociotechnical analysis framework used. The first version of the form was developed by the core analysis team. The sociotechnical dimensions were not changed by the team, but the added “Features and functions” dimension was retained, and the questions used in the Microsoft Excel template were clarified in many cases. The work process followed a weekly workshop format in which different framework dimensions were in focus and that lasted between February 2021 and April 2021. The draft template took form within a shared Google document, enabling collaborators in the participating countries to continuously offer feedback on the ongoing work. This format ensured that the items in the data collection form were relevant in the context of all involved countries. Finally, an analysis dimension called *metadata* was added during this iterative process, whereby information about the data collection itself—including the name of the system, the name of the researcher responsible for data collection, and information sources—could be noted.

After the first complete draft had been developed, a digital workshop was held in early May 2021 in which the core analysis team from Sweden (n=4) as well as representatives from Estonia (n=1), Finland (n=1), and Norway (n=2) participated. All participants are coauthors of this paper and are health informatics researchers with several years of experience following the implementation and subsequent use of the PAEHR system in their respective countries. The focus of the workshop was to discuss how to interpret the framework dimensions and uppermost to elaborate on and resolve some of the question formulations that had elicited many comments from other project partners in the shared document. After the 1-hour workshop, the template was refined, after which input was again sought from other project partners through email. After the last questions had been resolved, in October 2021, data collection could start based on the finalized template.

### Data Collection

Data collection was undertaken in 2 different phases. The first, longer phase started in October 2021 and ended in November 2021. One representative from each country, who also took part in the workshop where the data collection template was discussed, was assigned to be the main responsible data collector from their country, and communication among these representatives occurred continuously during the data collection period. Project participants from each country filled out a copy of the data collection form (Multimedia Appendix 1) for their main PAEHR systems and with the systems shown in Table 1 in focus.

**Table 1:** The studied national patient-accessible electronic health record (EHR) systems for patients’ web-based EHR access.

Country	System
Sweden	1177.se and Journalen [22]
Estonia	Digilugu [23]
Finland	Omakanta [24]
Norway	Helsenorge [25]

Some ambiguities were identified during the data collection, and these were handled through communication among the responsible researchers. When needed, the data collection master file was updated in accordance with agreed-upon solutions while making sure that everyone always used the question formulations in the latest version. After completion, all main responsible researchers from each country shared their filled-out forms in a shared folder for everyone to access.

After a preliminary walk-through of the collected data performed by the core analysis team, a need for further clarification was identified. In some cases, a few questions remained unanswered, and in other cases, the level of detail varied, requiring follow-up questions. When questions that the researchers from each country needed to elaborate on had been compiled, an additional and final data collection took place in December 2021. This shorter phase of data collection took place through email exchange between the leader of the core analysis team (JM) and the researchers responsible for data collection in each country. Multimedia Appendix 2 summarizes the sources of information that were used during the data collection in each country.

As a last step of data collection for this particular paper, a project representative from each involved country was tasked with collecting and summarizing information to enable a broader overview of national contexts than the developed data collection form could provide. In this final stage, information related to government structure, overall health care system, digital care organization, and steering of health information and communications technology (ICT) developments was gathered from each country. Information was gathered from national statistics, agency web pages, and local contacts within each health care system. Data collection for this study ended in November 2022.

### Analysis

When data collection was completed, the core analysis team began the analysis work. The first step was to copy all answers from the completed data collection forms into a shared analysis document. In this document, each dimension had its own sheet to simplify comparisons within each dimension. In each sheet, the questions from the data collection form were added as rows, and each PAEHR system was added as a column to create a matrix where each column included the answers related to a specific PAEHR system. Each answer, possibly in combination with an additional comment, was added to the corresponding cells in the matrix. Some examples are shown in the *Results* section. The content of the sheets was then compared across columns (countries) for all questions (rows) to identify similarities as well as aspects that are unique to the specific countries involved. In this step, representatives from Estonia, Finland, Norway, and Sweden were once again invited to discuss the identified similarities and differences.

### Ethical Considerations

The study presented in this paper is part of the larger NORDeHEALTH project, which received ethics approval from the appropriate national ethical bodies. This particular study did not involve human participants or sensitive personal data and only focused on contextual and technical details of PAEHRs; there were no specific ethical requirements that needed to be addressed.

## Results

### Final Data Collection Instrument

#### Overview

The final data collection form inspired by the sociotechnical dimensions in the framework developed by Sittig and Singh [17], including all updates made during the initial data collection round, can be found in Multimedia Appendix 1. Table 2 describes the overall content and structure of the form. The data collection form is the end result of the method development and is intended to enable sociotechnical comparisons of eHealth services for patients across countries, with a specific aim to compare PAEHR systems. It is important to note here that the form derived for this study is specifically aimed at comparison of PAEHR systems. For other types of eHealth services for patients, such as self-tracking applications, a different form may have to be derived through the process suggested in the *Methods* section.

**Table 2:** The sociotechnical framework dimensions of the final data collection form (following the work by Sittig and Singh [17])^a^.

Framework dimension	General description	Questions
Metadata	Information related to the data collection itself, such as the organizations and persons that are responsible for the data collection in the other dimensions, as well as the geographical and organizational context of the providers of PAEHRs^b^ in the country	1.1-1.6
Hardware and software computing infrastructure	The hardware and software required to run the applications; this also includes issues regarding information security, access control, and standards used	2.1-2.8
Features and functions	Important features and functions in the PAEHR service or in related services; some functions that may be considered external to a PAEHR in some contexts have been included, such as appointment booking, requesting renewal of prescriptions, and access to logs	3.1-3.14
Clinical content shared with patients	An inventory of which parts of a professional record are shared with the patients; examples are medications, laboratory test results of various kinds, images, and text notes	4.1-4.21
Human-computer interface	The “Human-computer interface” dimension captures information on studies conducted on the usability of the PAEHR service; it includes both the variables that have been measured and the results of measures from different stakeholders’ perspectives	5.1-5.10
People	“People” includes an overview of various characteristics of the population in the country, including language groups, educational levels, and internet use; it also includes user demographics of the PAEHR system	6.1-6.6
Workflow and communication	This dimension captures information focusing on collaboration and communication between health care professionals and patients and the explicit role of the PAEHR if identified and promoted in the country, possibly for distinct patient groups	7.1-7.11
The health care organization’s internal policies, procedures, and culture	An inventory of internal IT policy documents and managerial procedures that may influence the implementation and use of the PAEHR	8.1-8.6
National rules, regulations, and incentives	An inventory of national regulations that may facilitate or place constraints on the design, development, and implementation of the PAEHR	9.1-9.9
System measurement and monitoring	Collection of information on existing system measurement and monitoring programs to identify the use of the PAEHR system as well as individual functions; this also includes unintended consequences of the PAEHR service and other feedback to the national systems	10.1-10.5
Health care system context	The general context information about the health care system in the specific country where data are collected is recorded in this dimension; information about governance structure and EU^c^ membership as well as primary care organization and financing of health care is included in this dimension, and it is especially important if one performs comparisons across several countries	11.1-11.4

^a^The questions refer to the numbering in Multimedia Appendix 1.

^b^PAEHR: patient-accessible electronic health record.

^c^EU: European Union.

During the work process, some of the dimensions by Sittig and Singh [17] were adjusted, and some dimensions were added in response to identified needs that were not fulfilled by the original framework. The following dimensions were either updated or added (Table 2):

1. Metadata: this dimension was added during the work process to enable collection of information related to the data collection process itself. It stores general information that is important for keeping track, especially in large-scale projects.
2. Features and functions: we decided to use the existing framework dimension Clinical content (after renaming it—see the following item on this list) to describe the information to which patients or citizens are given access through the PAEHR (eg, laboratory test results, medications, and clinical notes). However, there is also variation in the functions that PAEHRs provide (eg, whether patients can comment or fill out forms). Considering the importance of these types of differences, we determined that a new dimension was warranted. Thus, this dimension ensured that the focus was not only on the clinical information that patients have access to in the PAEHR but also on the other important functions or features that they can use.
3. Clinical content shared with patients: in the original framework, this dimension was called “Clinical content.” The dimension was renamed to make it more PAEHR specific.
4. The health care organization’s internal policies, procedures, and culture: in the original framework, this dimension was more generally about policies, procedures, and culture. The decision was made to add “Health care organizations” as a specification of this dimension. This made it better suited for PAEHR analysis.
5. National rules, regulations, and incentives: in the original framework, this dimension was named “External rules, regulations, and pressures.” The redefined dimension puts the focus more on the national context in relation to rules and regulations, again to make it more suitable for PAEHR analysis.
6. Health care system context: this dimension was also added during the work process to understand the basics of the countries involved in the comparison. This understanding is needed when analyzing the data in the other dimensions as the national context, including health care system financing and steering of health ICT system development, has an effect on all the other dimensions. It is especially important to gather general data about the national context when comparing across several countries. This dimension was added to the final data collection form after the information had been collected in each country, and hence, it was added in response to a need that the data collection form used did not fulfill.

#### Identified Clusters of Dimensions

As the collected material is extensive, the dimensions were clustered to enable more manageable partitions of the data set. Dimensions that had similar focuses were grouped together. Textbox 2 presents the 4 resulting clusters. The *Contextual factors* *enabling PAEHR access and use* cluster gathers dimensions focusing on the user base as well as technical and governmental prerequisites for PAEHR access and use. The added *Metadata* dimension is also included in this cluster as it is a prerequisite for the type of comparative studies that we have conducted, and it also includes contextual information. The *Features and content* cluster includes dimensions focusing on what is actually offered to patients in the PAEHR, that is, the content (eg, clinical notes, test results, and images) and functions (eg, secure messaging and prescription renewal) that are provided to patients. The *Evaluations of human-computer interaction and use* cluster includes the dimensions focusing on how one measures and evaluates PAEHR use, as well as the results of such evaluations. Both internal service provider evaluations and external evaluations are included to provide a broad coverage. Finally, the *National and local policies, regulations for use, promotion, workflow, and communication* cluster includes dimensions focusing on laws and regulations; the focus in this cluster is also more on health care professionals and their relationship to PAEHRs than on the patients.

**Clustering of the sociotechnical framework dimensions used in the reporting of the findings.**

**Contextual factors enabling patient-accessible electronic health record access and use**

- Metadata
- Hardware and software computing infrastructure
- People
- Health care system context

**Features and content**

- Features and functions
- Clinical content shared with patients

**Evaluations of human-computer interaction and use**

- Human-computer interface
- System measurement and monitoring

**National and local policies, regulations for use, promotion, workflow, and communication**

- Workflow and communication
- The health care organization’s internal policies, procedures, and culture
- National rules, regulations, and incentives

In the article series about the results of the sociotechnical analysis, each article focuses on different clusters from Textbox 2. This paper has the *Contextual factors enabling PAEHR access and use* cluster in focus; hence, the dimensions belonging to this cluster were considered in detail, and the results of the data collection in relation to those dimensions are presented in the following sections.

### Results From the Sociotechnical Comparison of the “Contextual Factors Enabling PAEHR Access and Use” Cluster

In this section, the results gathered by means of the derived sociotechnical data collection form, with a focus on contextual factors, are presented.

#### Metadata

The metadata dimension, which was not part of the original sociotechnical framework presented by Sittig and Singh [17], includes basic information related to the data collection process itself and the system in question. Collected information about the PAEHR systems is presented in Table 3. Differences can be observed regarding the type of provider responsible for sharing EHR services in each country. In Sweden, the responsible provider is a publicly owned company, Inera AB, whereas the responsible providers in the other countries are institutions or ministries. In Sweden, Finland, and Estonia, the PAEHR service is national; however, in Norway, the studied service, Helsenorge, is only used for PAEHRs in 3 of 4 regions. All countries only provide 1 national PAEHR service (Table 3); however, local PAEHRs can exist in parallel with the national PAEHR. In all studied countries, health information from different sources is collected in a single PAEHR.

**Table 3:** Data collected for the items of the Metadata dimension by country^a^.

	Sweden	Norway	Finland	Estonia
Name of the national PAEHR^b^ service	1177 Journalen^c^	Helsenorge	Omakanta	Digilugu
Responsible provider	The Swedish eHealth organization Inera AB	The Norwegian Directorate of eHealth and Norwegian Health Network	The Finnish Social Insurance Institution (Kela)	The Estonian Ministry of Social Affairs (and the Health and Welfare Information Systems Centre and the National Institute for Health Development)
Geographic area	National	3 of 4 health regions in Norway	National	National
Number of national PAEHRs that 1 patient can have	1	1	1	1

^a^The items shown in the table correspond to questions 1.1 to 1.4 in the data collection form (Multimedia Appendix 1). Question 1.6 regarding information sources is answered in Multimedia Appendix 2.

^b^PAEHR: patient-accessible electronic health record.

^c^A total of 2 different Swedish systems (1177 and Journalen) were analyzed. As 1177 includes Journalen (which presents most of the PAEHR information) as well as some other related features, the results from the 2 systems are merged here. In cases in which, for example, the setup of Journalen differs from that of the rest of 1177, the differences will be highlighted.

#### People and Demographics

This dimension was more complex than the others covered in this paper as demographic data were not available from the same time intervals and age intervals in the different countries. In addition, some of the countries did not have user group statistics of the PAEHR available. Hence, a complete comparison across all countries cannot be made, and as a consequence, results will only be summarized at an overall level in this section.

Sweden has the highest number of inhabitants (10.2 million) of the countries compared, and Estonia has the smallest (1.3 million). Norway and Finland have 5.4 and 5.5 million inhabitants, respectively. Thus, there are large differences in population size among the countries. In addition, there are large differences regarding the proportion of immigrants in the countries, with the lowest proportion (9%) in Finland and the highest proportion (31% non-Estonians) in Estonia. In Norway and Sweden, the proportion is approximately 20%. Data on internet use among the populations of the 4 countries were available from 2019 in Finland and 2020 in Estonia, Norway, and Sweden. The data showed that a high proportion of the populations were internet users, with the highest number in Norway (98%) and the lowest in Estonia (89%). Data from all countries showed a general trend of increasing internet use. Estonia and Norway lack statistics on the use of PAEHRs in different user groups (such as age, gender, and profession). In Finland, statistics on PAEHR use in different age groups are collected, with the highest number of users being aged 36 to 50 years (94%) and 18 to 35 years (93%). In Sweden, data are collected for the PAEHR service Journalen when it comes to age intervals and gender. The service is used most frequently by individuals aged 20 to 29 years and 30 to 39 years, and slightly more female (53%) than male (47%) individuals use it. Educational levels are comparable across countries, with the vast majority of the population reaching upper secondary education. The highest proportion with at least 3 years of higher education can be found in Sweden (37%), and the lowest proportion can be found in Finland and Estonia (23%). No statistics from any of the countries could be found regarding the proportion of the population that prefers an interpreter in their contacts with health care.

#### Hardware and Software Computing Infrastructure

This dimension includes several important contextual factors that are presented in Table 4. Among the compared countries, 2 different ways of storing data are represented. In Norway, Finland, and Sweden, data are stored in local EHRs and then extracted and presented in the PAEHRs at runtime. For Digilugu in Estonia, centralized storage is used. In most of the studied countries, data are provided to the PAEHR from both private and public providers and from both primary and secondary care. The exception is Helsenorge in Norway, where only secondary care provides data to the PAEHR. Although all health care personnel in Norway are obliged to provide data to the EHR, it is decided on a regional level whether these data should be electronically accessible in the PAEHR. There are clear similarities among the compared countries when it comes to access points, enrollment, and authentication—all countries use 1 national access point, and each patient of a connected provider automatically receives a PAEHR. Authentication in all countries is made possible through a national electronic ID. Web browsers as well as mobile-adapted web browsers can be used by patients in all studied countries to access the PAEHR. In Norway and Sweden, there is also the possibility of using apps for iOS or Android. There are also some differences regarding how data are provided to the PAEHR. In the case of 1177, Omakanta, and Helsenorge, data are automatically linked to the source EHRs at runtime when a patient logs in to the system. In Digilugu, on the other hand, data are either automatically uploaded from source EHRs to a central server or manually uploaded from the EHR by a health care professional. Hence, in Estonia but not in the other studied countries, it is possible for a health care professional to add content to the PAEHR specifically.

**Table 4:** Data collected for the items of the Hardware and software computing infrastructure dimension by country^a^.

	Sweden (Vårdguiden 1177)	Norway (Helsenorge)	Finland (Omakanta)	Estonia (Digilugu)
Centralized or distributed data storage?	Data stored in local EHRs^b^	Data stored in local EHRs	Centralized storage	Centralized storage
Who provides data to the PAEHR^c^?	Private and public providers and primary and secondary care	Public providers and secondary care	Private and public providers and primary and secondary care	Private and public providers and primary and secondary care
One national access point per patient portal to the PAEHR?	Yes	Yes	Yes	Yes
How is enrollment done?	Each patient of a provider automatically receives a PAEHR	Each patient of a provider automatically receives a PAEHR	Each patient of a provider automatically receives a PAEHR	Each patient of a provider automatically receives a PAEHR
How are users authenticated?	Patients use a national electronic ID of some type	Patients use a national electronic ID of some type	Patients use a national electronic ID of some type	Patients use a national electronic ID of some type
What technical platform can the patients use for access?	Web browser, mobile-adapted web browser, app for iOS, and app for Android	Web browser, mobile-adapted web browser, app for iOS, and app for Android	Web browser and mobile-adapted web browser	Web browser and mobile-adapted web browser
How are data provided to the PAEHR?	Automatically linked to source EHRs at runtime	Automatically linked to source EHRs at runtime	Automatically linked to source EHRs at runtime	Automatically uploaded from source EHRs to a central server and manually uploaded from EHRs by a health professional
Are international standards (eg, FHIR^d^, openEHR, and ISO^e^ 13606) used in the interface between local EHRs and the PAEHR?	A national architecture for information services exists that defines information content that is not expressed in any international standard. However, many standards are used to build the integration. International terminologies are used for some aspects, such as *ICD-10*^f^ for diagnosis and ATC^g^ for class of medicinal product.	IHE^h^ XDS^i^ and IHE plus national information structure standards	National profiles of information content expressed in various HL7^j^ syntaxes—(HL7 version 3: CDA R2^k^ and HL7 FHIR) plus a number of modern web-based service standards for exchange and information security	National profile of old-type HL7 standards, such as HL7 version 3 and HL7 CDA R2 combined with international terminologies such as *ICD-10*, ATC, and LOINC^l^

^a^The questions correspond to questions 2.1 to 2.8 in the data collection template (Multimedia Appendix 1, where all response options can also be found).

^b^EHR: electronic health record.

^c^PAEHR: patient-accessible EHR.

^d^FHIR: Fast Healthcare Interoperability Resources.

^e^ISO: International Organization for Standardization.

^f^ICD-10: International Classification of Diseases, 10th Revision.

^g^ATC: The Anatomical Therapeutic Chemical Classification System.

^h^IHE: Integrating the Healthcare Enterprise.

^i^XDS: Cross Enterprise Document Sharing.

^j^HL7: Health Level Seven.

^k^CDA R2: Clinical Document Architecture Release 2.

^l^LOINC: Logical Observation Identifiers Names and Codes.

#### Health Care System Contexts

During the analysis of the collected data, it became clear that it is necessary to provide basic descriptions of the health care system contexts for readers who are unfamiliar with them. Therefore, a summary of the most essential information for understanding the PAEHR context is provided in Textbox 3. The full data are provided in Multimedia Appendix 3.

**Data collected for the items of the “Health care system context” dimension by country. The aspects considered correspond to questions 11.1 to 11.4 in the data collection template (Multimedia Appendix 1). For these items, there were only free-text responses.**

**Governance structure**

- Finland
  A parliamentary republic, which is a member of the European Union (EU)
  There are 3 levels of government: state, regions (19), and municipalities (310)
- Estonia
  A parliamentary republic, which is a member of the EU
  There are 3 levels of government: state, counties (15), and municipalities (79)
- Norway
  A constitutional monarchy, which is not a member of the EU (but a member of the European Economic Area)
  There are 3 levels of government: state, counties (11), and municipalities (356)
- Sweden
  A constitutional monarchy, which is a member of the EU
  There are 3 levels of government: state, regions (21), and municipalities (296)

**General health care system financing**

- Finland
  Municipalities are responsible for providing health care that is financed through local tax.
  Every resident in the country is entitled to health care services from the tax-funded system (funds from the 3 levels of government and a small private sector). Municipal authority hospitals provide specialist care, and privately owned hospitals supplement with, for example, day surgery.
- Estonia
  The health care is organized in 3 levels—primary or family care, specialist care, and nursing care
  Payroll tax covers 78% of all expenditures. The Estonian Health Insurance Fund (EHIF) is the sole provider of universal health coverage. The EHIF covers approximately 95% of the population; the rest is covered by the Ministry of Social Affairs. The ministry also has a main responsibility.
  All health care providers are independent entities; family physicians are local entrepreneurs in private companies.
- Norway
  The health care is organized in 2 levels—primary care (municipalities) and specialist care (the state). Specialist care is divided into 4 health regions and provides specialist care (mainly hospital based) and ambulance service. The state financing is channeled through the health regions.
  The tax-funded system covers health care for all citizens.
- Sweden
  The health care is organized on 3 levels—the state, regions, and municipalities. The regions with their own elected parliaments decide on regional tax, which provides the major financing for health care. National tax funds also constitute large parts of regions’ and municipalities’ health care budgets. Home care is funded through local municipality taxes.
  The tax-funded system covers everyone, including recent immigrants. It is free for inpatient care, and outpatient care has a low cost. Most of the care is operated by regions, but especially primary care and some specialist services are performed by private companies under contracts with the regions. The private share varies a lot among regions, with the largest private providership in the capital region where also a small private part exists independent of the public system that is financed mainly through optional insurance.

**Primary care**

- Finland
  These are municipality-arranged services at municipal health centers.
  They include population health monitoring and the promotion of well-being and health as well as prevention, diagnostic services, and treatment.
- Estonia
  Every EHIF-insured individual (and every Estonian resident) is assigned to a personal general practitioner (GP), who is the first level of contact. The insured individual can choose the GP. Approximately 70% of GPs are in solo practices. Practice lists cover the entire population. There are no copayment fees for primary care services in the EHIF package.
  The GP is the main point of contact for health benefits; they are expected to manage most patient pathways and to refer patients to specialist care or long-term nursing care and rehabilitation.
  Provides >50% of ambulatory care visits.
- Norway
  Services are arranged by the municipalities based on the GP scheme. All citizens have the right to register with a GP of their choice. There are approximately 5000 GPs in total.
  The GPs are the first line of contact; they coordinate care, are gatekeepers for welfare goods, and manage referrals to specialist care.
  Municipalities also offer, for example, emergency care, home care, and rehabilitation services.
- Sweden
  Services are arranged by the regions. Each citizen is connected to a primary care team (often physicians, nurses, psychologists, and physiotherapists), which is the first line of contact. There are approximately 900 primary care centers, between 10% and 50% private depending on the region. Primary care is never provided by single physicians. Care centers are relatively large, with 2 to 10 physicians and a total staff of 20 to 80 people. Organization differs somewhat among regions.
  Primary care handles many issues directly but may also refer to specialist inpatient or outpatient clinics. They also work with health promotion and preventive care (eg, vaccination) and maternity and child care and are the direct clinical contact of the municipalities’ home health care.

**Steering of health information and communications technology (ICT) development**

- Finland
  The national solution (Omakanta) is provided by the Social Insurance Institution (Kela). Private and public providers often also provide local portals with no connection to Omakanta.
  Decisions on regional health ICT systems are often made by municipalities or hospital districts. Health Village (portal implemented by all university hospitals) and Omaolo (municipality collaborative) are national initiatives.
- Estonia
  There are 2 main domains: Central databases, services, and applications: the National Health Information System (central database and services governed by the Ministry of Social Affairs) and the database of the EHIF are the major components. Databases and applications of health care stakeholders: electronic medical records (EMRs) and health information systems (HISs) of care facilities, as well as applications provided and maintained through private companies, are the major components. EMRs and HISs need to comply with central systems and legal regulations on, for example, data sharing.
- Norway
  The Directorate of eHealth was established in 2016 and is responsible for implementing national policy and steering and coordinating eHealth initiatives with stakeholders. They are generally responsible for the work in national eHealth programs, including the national portal Helsenorge. Since 2020, the Norwegian Health Network has had the responsibility to develop and operate the portal. It is the health regions’ decision whether to use the national offered health portal for patient-accessible electronic health records (PAEHRs) in their region.
- Sweden
  Major decisions are made by the 21 autonomous regions; hence, there is not much national coordination. However, collaborations on common procurement of electronic health record (EHR) systems among regions, as well as among secondary care, primary care, and municipalities in some larger regions, have happened recently (Cambio COSMIC is procured by 17 of 21 regions).
  Inera (subsidiary of the Swedish Association of Local Authorities and Regions) supports regions with ICT development and interoperability issues. Inera manages the 1177 patient portal, where Journalen resides.
  The eHealth authority (under the Ministry of Health and Social Affairs) manages electronic prescriptions of medicinal products. They hold the prescription database and link together all pharmacies.

## Discussion

### Aims and Motivation

The study presented in this paper had two main aims: (1) to develop a detailed collaborative method suitable for cross-country sociotechnical analyses of eHealth services for patients’ access to their records and (2) to illustrate the data collection method by comparing results regarding contextual factors enabling PAEHR access and use in 4 European countries. Starting from a sociotechnical analysis framework developed by Sittig and Singh [17] with an intention to produce a detailed data collection template for cross-country sociotechnical comparisons, a collaboration method was developed and implemented. The resulting data collection instrument was then used in a case study where a cross-country analysis was conducted based on collected data from Estonia, Finland, Norway, and Sweden.

This sociotechnical analysis is valuable because, while innovation in PAEHRs is important, there must be a concomitant, ongoing focus on how these tools are integrated into health systems. Digital innovations can fragment care or risk not being used, and examining the social and technical factors pertaining to PAEHRs in different settings is crucial. This will help us understand potential nonadoption; abandonment; and the effects of using such tools in health care on a wide range of stakeholders, including patients and clinicians.

### Method Development

Developing a method that involved collaboration among knowledgeable representatives from all countries involved was a necessity for arriving at the desired result—a data collection instrument that enabled a detailed cross-country sociotechnical system comparison. Earlier research based on more limited sociotechnical comparisons has shown that PAEHR systems differ greatly across countries, mostly due to differences in health care policy, underlying care structures, and health ICT initiatives [3]. To be able to develop a data collection instrument that both covers important aspects of the investigated countries and systems and is relevant in the respective health care system contexts, it was deemed of high importance to involve experts from each country.

The NORDeHEALTH project participants were not experts on every aspect covered, but they had established connections with relevant health authorities and development companies that could provide necessary information through documents or in-person communication. These contacts were necessary for the success of the study, and hence, the continuous contact with these external national agencies and companies was a vital part of the developed collaboration method. On the basis of the experience with the method development, we argue that these external connections need to be established before doing similar research in the future.

The sociotechnical analysis framework by Sittig and Singh [17] on which this work was based was general and theoretical in the sense that dimensions but not any specific data collection items were presented. The framework was also aimed toward sociotechnical health systems in general and not PAEHR systems or even eHealth services for patients in particular. Hence, a big part of the method was devoted to developing a data collection instrument that would work in this specific context—a cross-country analysis of PAEHR systems in the 4 involved countries. An inevitable consequence of this is that the data collection instrument developed in this study cannot be used to enable sociotechnical analysis of other kinds of health care systems. This being said, a similar method can be used to develop other data collection instruments that include items for the different framework dimensions that are better suited to other kinds of health care systems.

The framework by Sittig and Singh [17] has indeed been adapted for sociotechnical analysis of health care technology systems but not for cross-country comparisons. As a consequence, an important part of this work was to amend it for the purpose of comparing health care systems across countries. In total, 2 dimensions were added to the framework for this purpose—metadata and health care system context. It is beneficial to include the *Metadata* dimension in any kind of sociotechnical analysis, but we argue that it is especially important to include this dimension when comparing data from different countries with their own systems, responsible data collectors, and information sources.

The collection of data for the *Health care system context* dimension occurred after the data collection that was based on the developed data collection instrument. After data collection, this dimension was added as a last step of the iterative development of the method and the data collection instrument in line with the design science research approach followed in this study. As earlier research has shown that PAEHR systems vary across countries due to contextual factors [3], this dimension is of high importance to consider in a cross-country comparison of the kind carried out in this study. The national setup of primary and specialist care, as well as general digital care infrastructure and steering of health ICT development, can affect the results of all other framework dimensions. Hence, we argue that this dimension is necessary to include in these kinds of studies.

### Country Comparison

The sociotechnical cross-country comparisons regarding contextual factors brought to light several differences and similarities among the involved countries. *Hardware and software computing infrastructure* was the dimension in which most of the similarities could be found. However, it is noteworthy that the use of standards differs considerably across countries, which could potentially give rise to interoperability issues in case, for example, the Nordic countries were to move toward a joint service. It is also of interest to note that physicians in Estonia manually upload some content to the PAEHR, whereas Sweden and Norway only use the automatic link to EHRs at runtime. In Finland, content is also uploaded to the centralized PAEHR but by technical or administrative staff rather than clinicians. This means that health professionals in Estonia can be said to be active users of the PAEHR services. There are some risks associated with being an active user. Aside from the fact that the workload of health care professionals increases, there is also a risk that the personal opinions of health care professionals may affect their willingness to share information and, consequently, the patients’ potential to access their information. Overall, the level of engagement of health care professionals affects whether patients will be able to access some information. In Norway and in some regions in Sweden, patients can only see notes that are signed by a health care professional. Some regions in these countries also have a default delay in publishing notes to patients regardless of whether they are signed. This makes it possible to hide information from patients for some time, for example, by not signing a note.

When it comes to user demographic information, similarities could also be found, but it was difficult to compare data due to differences among the statistical information that the countries provide and how these data are presented. In these kinds of analyses, it is important to be able to compare user statistics on how different groups use the PAEHR services, and this comparison was not possible as the statistics covered different user groups in Sweden and Finland. Estonia and Norway did not (at the time of data collection) collect statistics on individual PAEHR use. The difference in available national statistics on demographic use clearly shows the need to collect user statistics preferably based on comparable user groups and age intervals. This would aid future comparisons as well as evaluations of PAEHR services within countries.

In the *Health care system context* dimension, several differences could be found regarding health care system setup and financing. While all involved countries have 3 levels of government (state, regions or counties, and municipalities), there are differences in which levels provide most of the funding and are responsible for different levels of care. While the regions are responsible for most health care services in Sweden, the responsibility is more divided in the other countries. Differences are also found regarding steering of health ICT development. While, in most countries, the national PAEHR solution is managed by government-owned companies or insurance institutions, there are differences in other areas of health ICT.

### Strengths and Limitations

To our knowledge, this kind of detailed comparison among PAEHR systems in different countries is novel. While Essén et al [3] compared PAEHR systems in 10 different countries, including Estonia, Finland, Norway, and Sweden, their comparison focused on the effect of differences in regulations on a few aspects of PAEHRs. Our study expands this research by using a complete sociotechnical framework as a base, enabling more detailed comparisons and a more in-depth analysis of similarities and differences across countries. This kind of comparison is important if we want to understand the effects of contextual factors on the realization of PAEHR systems and will also make it possible for systems to learn from each other. This learning takes place not only when analyzing the results from the data collection but also when carrying out the developed collaboration method.

This study used a specific sociotechnical framework as a base, and even though the framework by Sittig and Singh [17] is tailored toward health systems, it risks constraining the focus to certain dimensions. There are other sociotechnical frameworks that could have been used instead. This being said, the involved researchers did add and change some dimensions to better fit PAEHR systems in particular and the cross-country comparison. Hence, even though a specific framework was used as an important base, it did not completely constrain the focus.

The number of countries involved—and, hence, the number of compared systems—was fairly limited, especially considering the fact that there are several clear similarities among the Nordic countries when it comes to national contexts. Comparisons involving more diverse countries should be conducted when validating the method.

### Future Work

This paper presented the results of a sociotechnical cross-country comparison among PAEHR systems in Estonia, Finland, Norway, and Sweden related to contextual factors. The dimensions that were in focus in this paper cover 4/11 of the total dimensions in the data collection instrument. Thus, there are more in-depth comparisons being made based on the data collected in this study. In future work, more dimensions will be included, and the results will be published in subsequent papers.

In future research, it would also be beneficial to increase the number of countries to be compared. The next step in this direction could be to extend the analysis conducted by Essén et al [3] by using the framework by Sittig and Singh [17] and the collaboration method presented in this paper. This would not only include a comparison of PAEHR systems from more countries with larger differences in national contexts, but it would also validate the method in a more complex study. These kinds of more expanded comparisons could also use other frameworks as their base. It is also important to note here that digital forms were not developed in this study as the number of systems to be included in the data collection in the 4 involved countries was fairly limited and, hence, the statistical analysis made possible in many digital tools would not add any value. Instead, the data collection was prepared as a Word template to be filled out digitally. In future research including more countries and systems, it would be well worth introducing digital forms and statistical analyses.

The study presented in this paper only focused on PAEHR systems. Even though these systems are in increasing focus in research nowadays, there are many more types of eHealth services for patients as well as other types of health care systems. In future research, we would like to see this collaboration method used for developing data collection instruments related to other kinds of health care systems, such as apps for self-tracking or systems for video visits.

Future research could also usefully focus in greater depth on particular sociotechnical dimensions of PAEHRs in different countries. For example, regarding human-computer interfaces, investigators might explore the highly specific design features of portals, which may augment psychological dispositions to access health information, or (possibly more importantly) offer feedback to providers.

### Conclusions

In this work, we aimed to develop and test a method that would enable cross-country sociotechnical analysis of PAEHRs in 4 countries where PAEHR use has reached maturity: Estonia, Finland, Norway, and Sweden. The main artifact produced during this work was a sociotechnical data collection template that was based on a sociotechnical framework for studying eHealth services derived by Sittig and Singh [17]. The data collection template not only considers the dimensions in the framework by Sittig and Singh [17] tailored for single-system analysis but also includes parts deemed necessary for cross-country comparisons of several systems. Close collaboration during the process among researchers from all involved countries ensured relevance in all settings.

The data collection template was tested through data collection in the 4 countries and a sociotechnical analysis. Results indicated several important similarities and differences among the 4 countries, clarifying that the process followed and the data collection template enabled an in-depth cross-country sociotechnical analysis of PAEHR systems. This paper presents the first part of the results from the sociotechnical analysis—similarities and differences among contextual factors—and companion articles will delve deeper into the remaining parts.

## Appendix

Multimedia Appendix 1 Final data collection instrument.

Multimedia Appendix 2 Information sources.

Multimedia Appendix 3 Descriptions of health care system contexts.

## Abbreviations

EHR: electronic health record
ICT: information and communications technology
PAEHR: patient-accessible electronic health record
RQ: research question

## Data Availability

The data sets generated and analyzed during this study are not publicly available due to the fact that additional publications based on this data set are being produced, but are available from the corresponding author on reasonable request.
