# Supplementary material for: Sociotechnical Cross-Country Analysis of Contextual Factors That Impact Patients’ Access to Electronic Health Records in 4 European Countries: Framework Evaluation Study
Source: J Med Internet Res. 2024 Aug 26;26:e55752. doi: 10.2196/55752 (PMC11384177; doi:10.2196/55752)
Supplement: Multimedia Appendix 1 [file jmir_v26i1e55752_app1.doc]

**NORDeHEALTH: Socio-technical data collection form**

| 1. **Metadata for the Questionnaire answers**   **In this study we are investigating digital health services available to all inhabitants in a geographical area (national for all of a country or regional). The main interest is in services which give a patient or next-of-kin access to a health record written by care professionals but we are also interested in services that after authentication provide certain other functions, such as a secure communication channel to a healthcare unit or professional tools for a patient written personal health record. We are not including services that are restricted to a certain patient group (with a specific diagnosis) and we are not including access to real time communication systems for triage, chat or video meetings. Also services that provide general information on health or healthcare which is not tailored to the individual is out of scope.**  **Note there will be one answer form per service. Do not mix different systems/services in the responses.** | | |  |
| --- | --- | --- | --- |
| **Question number** | **Question text** | **Alternative answers** | **Comment-discussion and REFERENCES** |
| Q1.1 | Name of the national PAEHR service | Answer: <txt> |  |
| Q1.2 | Responsible provider of the PAEHR | Answer: <txt> |  |
| Q1.3 | Geographic area for the PAEHR | Answer: <txt> |  |
| Q1.4 | How many national PAEHRs can one patient have? | [] One  [] Several |  |
| Q1.5 | Who is providing the data collection within NORDeHEALTH  (NORDeHEALTH data collector)? | Answer: <txt> |  |
| Q1.6 | Information source(s) persons or documents | Answer: <txt> |  |
|  |  |  |  |
| **2. Hardware and Software Computing Infrastructure**  *Focuses only on the hardware and software required to run the applications* | | |  |
| Q2.1 | Centralized or distributed data storage? | [] Centralized storage  [] Distributed storage  [] Data stored in local EHRs (and extracted to Journalen) |  |
| Q2.2 | Which healthcare providers are providing data  to the PAEHR? | [] Private providers  [] Public providers  [] Primary care  [] Secondary care  [] Social care providers |  |
| Q2.3 | Is there one national access point/patient portal to the PAEHR? | [] Yes  [] No  Comments: |  |
| Q2.4 | How is enrollment done? | [] Each patient of a provider automatically gets a PAEHR  [] Each patient has to apply online to get a PAEHR  [] A Health professional has to create the PAEHR for each patient |  |
| Q2.5 | How is authentication of users made? | [] Subjects have a password  [] Subjects use a national electronic ID of some type  [] Subjects need a biometric ID |  |
| Q2.6 | What technical platform on the device can the patients use for access? (One or several answers) | [] Web browser  [] Mobile adapted web browser  [] App for IOS device  [] App for Android device |  |
| Q2.7 | How is data provided to the PAEHR? | [] Automatically linked to source EHRs at runtime  [] Automatically uploaded from source EHRs to a central server  [] Manually uploaded from EHR by a Health Professional  [] Manually written to PAEHR by a Health Professional  [] Manually written by the patient |  |
| Q2.8 | Are international standards (e.g. FHIR, openEHR, 13606) used in the interface between local EHRs and the PAEHR? | [] Yes                              If yes, which: <txt>  [] No  Comments: |  |
| **3. Features and Functions**  *Important features and functions in the PAEHR service, or in related services. Since there is not yet a strict definition of what functionality is included in a PAEHR or not, we have included functions that may be considered external to a PAEHR in some contexts.* Note there will be one answer form per service. Do not mix different systems/services in the responses*.* | | |  |
| Q3.1 | Does the service include appointment booking? | [] Yes, in national solutions,  [] Yes, in local solutions  [] Yes, but...  [] No  Comments: |  |
| Q3.2 | Does the service include a function to request renewing of prescriptions? | [] Yes  [] No  Comments: |  |
| Q3.3 | Does the service include a function for secure messaging from the Patient to the Professional? | [] Yes  [] No  Comments: |  |
| Q3.4 | Does the service include a function for secure messaging from the Professional to the Patient? | [] Yes  [] No  Comments: |  |
| Q3.5 | Does the service include a function for patients to annotate an item in the PAEHR? | [] Yes  [] No  Comments: |  |
| Q3.6 | Does the service allow search functions for patients in the PAEHR documents? | [] Selection of dates  [] Selection of part of the clinical record  [] Selection of individual laboratory results  [] Free text search  [] No search or selection function  Other (specify): <txt> |  |
| Q3.7 | Does the service allow filtering functions for patients in the PAEHR documents? | [] Selection of dates  [] Selection of part of the clinical record  [] Selection of individual laboratory results  [] Free text search  [] No search or selection function  Other (specify): <txt> |  |
| Q3.8 | Does the service include a function for exporting data? | [] Yes  [] No  Comments: |  |
| Q3.9 | Does the service include a function for printing? | [] Yes  [] No  Comments: |  |
| Q3.10 | Is there a notification feature signaling updates to the health record? | [] Yes via sms  [] Yes via email  [] Yes via frontpage in the portal  [] Yes (only)  in the PAEHR  [] No  Comments: |  |
| Q3.11 | Does the service allow patients to control authorizations (who can see what)? | [] Block patient's own access  [] Patients can name a proxy for access  [] Control access by professionals to a shared record  [] No authorizations via the PAEHR service  Other (specify): <txt> |  |
| Q3.12 | Does the service include a log list, showing who has accessed the medical record? | [] Yes  [] No  Comments: |  |
| Q3.13 | Can the patient access functions for decision support? | [] Yes                           If yes, which: <txt>  [] No  Comments: |  |
| Q3.14 | Does the service allow the patient to view scheduled appointments in the future? | [] Yes  [] No  Comments: |  |
| **4. Clinical Content Shared with Patients**  *Includes everything on the data-information-knowledge continuum that is stored in the PAEHR service and made accessible to patients.* | | |  |
| Q4.1 | Is a summary record provided?  A summary record is an overview of the patient record with most important facts to be known by all. It can include alert information (allergies etc) | [] Yes in PAEHR  [] Yes in portal  [] No  Comments:  (e.g. not in PAEHR but outside) |  |
| Q4.2 | Summary record: Provided by whom? | Answer: <txt> |  |
| Q4.3 | Summary record: Provided for what purpose? | Answer: <txt>  (e.g. for the primary care to get a quick overview of pat) |  |
| Q4.4 | Does the PAEHR provide a dedicated care plan? | [] Yes  [] No  Comments: |  |
| Q4.5 | Does the PAEHR provide diagnoses? | [] Yes (all)  [] Yes (current)  [] Yes separating chronic from temporary  [] No  Comments: |  |
| Q4.6 | Does the PAEHR provide laboratory results? (several answers possible) | [] Yes chemistry including point of care analyses  [] Yes microbiology  [] Yes immunology  [] Yes genetic information  [] No  Comments: |  |
| Q4.7 | Does the PAEHR provide a list of current medication? | [] Yes updated as agreed with the doctor  [] Yes in the form of the recently issued prescriptions  [] No  Comments: |  |
| Q4.8 | Does the PAEHR provide a list of historic prescriptions? | [] Yes  [] No  Comments: |  |
| Q4.9 | Does the PAEHR provide a list of prescriptions that may need a renewal? | [] Yes  [] No  Comments: |  |
| Q4.10 | Does the PAEHR provide a list of medications where there has been an adverse reaction? | [] Yes  [] No  Comments: |  |
| Q4.11 | Does the PAEHR provide a list of possible interactions or other warnings related to the current medication list? | [] Yes  [] No  Comments: |  |
| Q4.12 | Does the PAEHR provide a list of immunizations? | [] Yes  [] No  Comments: |  |
| Q4.13 | Does the PAEHR provide results of pathology reports? | [] Yes  [] No  Comments: |  |
| Q4.14 | Does the PAEHR provide results of physiology lab examinations (EKG, Echocardiograms, EEG etc) | [] Yes  [] No  Comments: |  |
| Q4.15 | Does the PAEHR provide results of point of care or home measurements? (length, weight, blood pressure, temperature, Oxygen saturation etc.) | [] Yes  [] No  Comments: |  |
| Q4.16 | Does the PAEHR provide imaging reports? (x-ray, MRI, or ultrasound) | [] Yes  [] No  Comments: |  |
| Q4.17 | Does the PAEHR provide images with viewing tools? | [] Yes  [] No  Comments: |  |
| Q4.18 | Does the PAEHR provide medical alert/critical information? | [] Yes in PAEHR  [] Yes in portal  [] No  Comments:  (e.g. not in PAEHR but outside) |  |
| Q4.19 | What type of notes does the PAEHR provide? (several answers possible) | [] Doctor’s notes from an outpatient visit  [] Doctor’s daily notes from a hospital stay  [] Nurses notes from outpatient visits  [] Nurses notes from hospital stays  [] Psychologist notes  [] Social worker notes  [] Maternity care information  [] Small children records  [] Psychiatrist notes  [] Discharge summaries (epicrisis)  [] Community care notes  [] Planning of joint care after discharge  [] Referrals  [] Referral responses and planning data  [] Surgery reports  [] Intensive care unit records |  |
| Q4.20 | What specific forms does the PAEHR provide | [] Patient self declaration of life style including        [] Tobacco        [] Alcohol        [] Exercise        [] Food  [] Health declaration for surgery  [] Health declaration for vaccination  [] Diabetes summary  [] Foot examinations  [] Psychiatric assessment scales (mini-mental, KEDS etc) |  |
| Q4.21 | Does the PAEHR provide access to Covid certificates? | [] Yes, to generate a certificate  [] Yes, to display a stored certificate  [] Yes, to download a generated certificate  [] No  Comment:  (e.g. type of certificate) |  |
| **5. Human Computer Interface**  *The usability of the PAEHR service.* | | |  |
| Q5.1 | What kind of evaluation/research of the services has been performed in your country? | [] Inspection methods (experts evaluate)     (explain: when, by whom, context): <txt>  [] Test methods (users evaluate)     (explain: when, by whom, context): <txt>  [] Self evaluation (Owner e.g. Inera makes own evaluations)     (explain: when, by whom, context): <txt> |  |
| Q5.2 | Is there continuous monitoring/evaluation of usability e.g. at defined intervals? | [] Yes [] No Comments: |  |
| Q5.3 | Which aspects of usability, (according to ISO 9241-11) have been considered in the usability evaluations? | [] Effectiveness  [] Efficiency  [] Satisfaction |  |
| Q5.4 | What were the results of the  usability studies? (Please, relate to the answers in Q5.1 and Q5.2) | Answer: <txt>  (If possible: add link/reference to results) |  |
| Q5.5 | What kind of international usability standards have been followed in the development? | Answer: <txt>   (Please specify which, when, and how these were met)  [] None |  |
| Q5.6 | Is the PAEHR supportive to users with cognitive and/or visual disabilities?  [e.g using WCAG 2.0 (ISO) Web Content  Accessibility Guideline and/or  in accordance with EN 301 549]  <https://www.digg.se/4a9ec3/globalassets/dokument/digital-tillganglighet/mall-for-tillganglighetsredogorelse-engelska.docx> | [] Yes      If yes, please specify which Accessibility Requirements and how these were met, e.g. Impaired vision and/or impaired cognitive function: <txt>    [] No  Comments: |  |
| Q5.7 | Is there a tailored design for adolescents? | [] Yes            If yes, describe the difference: <txt>  [] No  Comments: |  |
| Q5.8 | Does the PAEHR include support for different languages that are used in the country? | [] Yes            If yes, which languages are supported?: <txt>  [] No  Comments: |  |
| Q5.9 | The adoption to different languages apply to: | [] Navigation only  [] Content of the record including notes |  |
| Q5.10 | Does the PAEHR include features supporting orientation [website navigation]? | [] Yes            If yes, which kind of features?: <txt>  [] No  Comments: |  |
| **6. People**  *Represents the humans involved in all aspects of the implementation and use of the eHealth application, and how they experience the use. Please provide references to* ***data on population statistics*** *- and comment on their reliability!* | | |  |
| Q6.1 | Number of inhabitants in the country (in millions) | Answer: <txt> |  |
| Q6.2 | Proportion of inhabitants born in another country | Answer: <txt>  Comments: |  |
| Q6.3 | How many % of the population does currently have Internet access? | Current proportion: <txt>  Comment on statistics (e.g. year): <txt> |  |
| Q6.4 | Current use of the PAEHR in different groups (age, gender, profession, socioeconomic status) | Current proportions: <txt>  Comment on statistic: <txt>  [] Not applicable |  |
| Q6.5 | Current education level in the population (percentage) | [] Research, (third cycle) of higher education  [] Higher education, >3 years (second cycle-master)  [] Higher education <= 3 years (first cycle -bachelor)  [] Higher vocational education ( vocational diploma)  [] 12 years school -Upper secondary education  [] Elementary school  [] No formal education |  |
| Q6.6 | Proportion of population that prefer an interpreter in contacts with health care | Current proportion: <txt>  Comment on statistic: <txt> |  |
| **7. Workflow and communication**  *Focusing on collaboration and communication between different users, and assessing how well the eHealth application supports the current clinical workflow.* | | |  |
| Q7.1 | Is there a delay period before new information is visible in the PAEHR? | [] No, information is always available immediately when entered  [] Yes, information is only available after a certain period  [] Yes, specific data types are only available after a certain period  If yes, give the delay period and data types, if applicable: <txt>  Comments: |  |
| Q7.2 | What is the average or maximum time between event and availability of written notes (when records are dictated for transcription)? | Give examples from the studies found: <txt> |  |
| Q7.3 | Can the patient access unsigned /unvalidated notes? | [] Yes  [] Yes, with delay  [] No  Comment on decisions made: <txt>  (e.g. regional differences may occur?)  Other comments: |  |
| Q7.4 | Can the patient access unsigned/ unvalidated lab results? | [] Yes  [] Yes, with delay  [] No  Comment on decisions made: <txt> |  |
| Q7.5 | Is there any documentation, practice education or guidance for healthcare professionals on the usage of the PAEHR? | [] Yes              If yes, which type? <txt>  [] No  Comments: |  |
| Q7.6 | Is it possible for the patient to add information to the PAEHR (patient-created data)? | [] Yes              If yes, which type of information? <txt>  [] No  Comments: |  |
| Q7.7 | Is there a possibility to submit identified errors (as a quality control) as a comment from the patient to the clinical staff | [] Yes, within the PAEHR  [] Yes, as a messaging feature outside the PAEHR  [] No  Comments: |  |
| Q7.8 | Is it possible for health professionals to see that a patient has read information in the PAEHR? | [] Yes               If yes, how is reading confirmed?  <txt>  [] No  Comments: |  |
| Q7.9 | Can patients communicate with the healthcare professional (HCP) directly? | [] Yes  [] No  Comments: |  |
| Q7.10 | Can patients communicate with their HCP through a mediator (e.g. administrator/secretary) via the portal? | [] Yes  [] No  Comments: |  |
| Q7.11 | Can a conversation thread be maintained between the patient and their HCP? (as e.g. in a CMS, case management system) | [] Yes  [] No  Comments: |  |
| **8. Healthcare Organizations Internal Policies, Procedures, and Culture**  *Affect every other dimension in this model, since it includes any internal IT-policy documents and managerial procedures that may influence the implementation and usage of eHealth.* | | |  |
| Q8.1 | Are there any regional guidelines or recommendations related to the use of PAEHR? | [] Yes              If yes, please describe e.g. if is is an implementation of a national guideline: <txt>  [] No  Other comments: |  |
| Q8.2 | Is “the service” generally promoted to patients by the healthcare organisations? | [] Yes  [] No  Comments: |  |
| Q8.3 | Is “the service” generally promoted to HCPs by the healthcare organisations? | [] Yes  [] No  Comments: |  |
| Q8.4 | Do the healthcare organizations routinely inform patients about the PAEHR? | [] Yes  [] No  Comments: |  |
| Q8.5 | Do the health professionals routinely inquire of patients about their use of the PAEHR? | [] Yes  [] No  Comments: |  |
| Q8.6 | (How) Has the health professionals’ view of the PAEHR changed since it was implemented? | [] Yes  [] No  Comments: |  |
| **9. National Rules, Regulations,  and Incentives**  *External forces that facilitate or place constraints on the design, development, implementation, use, and evaluation of eHealth in the respective clinical settings.* | | |  |
| Q9.1 | Are there any national regulations related to PAEHR? | [] Yes      If yes, please specify which: <txt>  [] No  Comments: |  |
| Q9.2 | Are there any particular national  incentives for PAEHR implementation? | [] Yes      If yes, please specify: <txt>  [] No  Comments: |  |
| Q9.3 | Do the patients have the right to decide which information should be available in the summary record also accessible by health professionals? | [] Yes      If yes, specify which: <txt>  [] No  Comments: |  |
| Q9.4 | Can patients share all or parts of their PAEHR with a proxy of their choice? | [] Yes, only the whole PAEHR  [] Yes, the whole PAEHR or selected parts  [] No sharing allowed  Comments: |  |
| Q9.5 | Do legal guardians/parents have the right to access the childrens’ PAEHR? | [] Yes               If yes, comment on e.g. legal aspects and possible age limits when guardian can no longer access the children’s PAEHR: <txt>  [] No  Other comments: |  |
| Q9.6 | Which is the age limit for accessing your own PAEHR? | Age when access is granted: <number> |  |
| Q9.7 | Which is the model for healthcare funding? | [] Public or tax funding  [] Private or insurance funding  [] A mix of both public and private funding  Comments: |  |
| Q9.8 | What are the rules for inclusion of providers in the PAEHR services? | [] It is mandatory for all providers to offer the service  [] It is optional for providers to provide the PAEHR |  |
| **10. System Measurement and Monitoring**  *Focuses on the need for an effective system measurement and monitoring program to identify the availability of features and functions and how they are used, as well as expected outcomes and unintended consequences of the PAEHR service.* | | |  |
| Q10.1 | Are there any evaluations of the PAEHR? | [] Yes              If yes, please specify: <txt>  [] Yes, these are recurring  [] No  Comments: |  |
| Q10.2 | What were the patient’s views? | Answer: <txt> |  |
| Q10.3 | What were the professionals’ views? | Answer: <txt> |  |
| Q10.4 | Has usage statistics been used in monitoring the PAEHR? | [] Yes               If yes, please specify: <txt>  [] No  Comments: |  |
| Q10.5 | Is the usage data publicly accessible? | [] Yes, on a national level  [] Yes, on a regional level  [] No  Comments: |  |
| **11. Healthcare System Context**  *Focuses on general contextual information about the country, related to governance structure and healthcare organisation and financing. This dimension affects all other dimensions.* | | |  |
| Q11.1 | Description of the government structure and international relations | Answer: <txt> |  |
| Q11.2 | Description of the general healthcare system financing | Answer: <txt> |  |
| Q11.3 | Description of primary care organization | Answer: <txt> |  |
| Q11.4 | Description of the steering of health ICT development | Answer: <txt> |  |
