# Supplementary material for: Sociotechnical Cross-Country Analysis of Contextual Factors That Impact Patients’ Access to Electronic Health Records in 4 European Countries: Framework Evaluation Study
Source: J Med Internet Res. 2024 Aug 26;26:e55752. doi: 10.2196/55752 (PMC11384177; doi:10.2196/55752)
Supplement: Multimedia Appendix 2 [file jmir_v26i1e55752_app2.doc]

| **Country** | **Information sources** |
| --- | --- |
| Sweden (1177 Journalen) | Interviews with 3 representatives from INERA, the organization that manages the portal 1177.se and its service Journalen.  Interview with several specialists.  Publicly available information on 1177.se and Inera.se. |
| Estonia (Digilugu) | “Ten Years of the e-Health System in Estonia” - <http://ceur-ws.org/Vol-2336/MMHS2018_invited.pdf>  Patient Portal - <https://www.digilugu.ee/>  Online interview with key representative from the Health and Welfare Information System Centre in Estonia |
| Finland | Online interviews and several email clarifications with a specialist of Kela’s Kanta Services.  Publicly available information on Omakanta on [www.kanta.fi](http://www.kanta.fi/) |
| Norway | Webpages helsenorge.no, ehelse.no, nhn.no  Interviews with key representatives from University Hospital of North Norway (UNN) and Norsk Helsenett. |
