# Supplementary material for: Sociotechnical Cross-Country Analysis of Contextual Factors That Impact Patients’ Access to Electronic Health Records in 4 European Countries: Framework Evaluation Study
Source: J Med Internet Res. 2024 Aug 26;26:e55752. doi: 10.2196/55752 (PMC11384177; doi:10.2196/55752)
Supplement: Multimedia Appendix 3 [file jmir_v26i1e55752_app3.doc]

# Descriptions of healthcare system contexts

# Country: Sweden

## Government structure

Sweden is a constitutional monarchy with the official head of state now king Carl XVI Gustaf having only a symbolic role. The parliament has 349 seats.

Sweden has a strong tradition of local government with two levels below the national state with independently elected decision bodies and with the right to decide and collect taxes from their population. These are the 21 Regions with responsibility for public healthcare provision, regional transportation and some regional planning. There are three large regions: Stockholm 2,4 million, Västragötaland, 1,7 million and Skåne 1,4 million. Most of the regions have around 300 000 inhabitants.

There are 296 municipalities (“kommuner”) with very different sizes from Stockholm with 900 000 to 2800 in the smallest. The municipalities have about twice the budget size of the regions and are responsible for many issues including care for the elderly and those with functional impairments, schools, day-care of children, support of culture and local planning with roads, water and wastewater handling.

## Internationally

Sweden is a member of the European union and OECD[[1]](#footnote-2).

## General healthcare system

In Sweden the public healthcare system is very comprehensive and covers essentially everybody, even recently immigrated people and migrant workers from other European countries as well. It is organized with a regional responsibility of the regions. The regions provide health services free of charge as regards hospital inpatient care or at a low cost for an outpatient visit from 10-40 EUR. Approximately 90 % of all healthcare organizations are also operated by the regions, i.e. by the Public and on a non-profit basis; 10%  are run by private enterprises some by the doctors and in some cases other staff but in most cases larger even multinational companies own private healthcare enterprises, and here profit is accepted. However, most of this private sector operates under contracts from the regions in much the same way as region owned enterprises and the patient pays the same amount which is only a rather small nominal fee which has no connection to the real cost. The private and publicly owned healthcare organizations are paid from the regions for the visits and other services and in some formula often for the population or area responsibility. The local municipalities have some co-operation with their regions as regards health and care for the elderly but the general regulation states that municipalities do not perform qualified medical services and have no physicians employed. Their health records are not shared with the patients.

The regions and municipalities have a joint co-operation body called SKR or The Swedish Association of Local Governments and Regions. This is financed and controlled by the regions who give some money for joint development projects also supported by some state grants. This body works with attempted consensus and has no steering power over the local and regional bodies.

The central government provides requirements in general legislation and oversees quality. The national board of health also licenses certain health professional groups such as physicians, nurses and midwives and collects  highly detailed yearly  statistics. But they have no part in financing and have relatively little control of the activities of the regions and local municipalities.

## Primary Care

Primary care is a very essential part of the Swedish healthcare system and is available all over the country. It is sometimes called family medicine since it is supposed to be the first line of health services for all family members but it is by no means restricted to family issues and a very large proportion of Swedish households are single persons living on their own. The principle is that the citizens should always first see their primary care team unless obviously in need of hospital inpatient services. The primary care handles many health issues and takes care of long term follow up for most chronic diseases. When the patient is in need of special services the primary care physician makes a formal referral to the specialist in an outpatient clinic or if needed for hospital inpatient care as for a major operation etc. The specialists report back in a discharge summary from a hospital stay or otherwise for one or several outpatient visits. In this way the patient's primary care team (nurses, physicians and other professionals as physiotherapists and sometimes midwives or psychologists) should be able to follow their patient’s total health history.

Primary care centres not only take care of health problems when patients seek attention for certain symptoms, PHCC works actively with health promotion and various preventive measures from immunizations to stop smoking aid, control of blood pressure and even weight.

There are >900 Primary Healthcare Centres and 10-50 % of these are run by private companies, with the highest frequency in the three large cities. The 21 independent regions in Sweden have different political parties in control, and how services are organized may vary a little, for example the number of private PHCCs.

Most primary care centres have special units for maternity care MVCs and for small children, BVCs.

## Steering of Health ICT developments

Since most of the care system is financed and ruled by the 21 regions, these are also the bodies where major decisions about development take place. Compared to some other countries there has thus been little national co-ordination of developments and procurements of ICT for health. So, the major decisions on regional Electronic Health Systems have been made independently. However, recently there has been a strong concentration trend where regions in groups have agreed to do common procurements and soon 17 of the 21 regions will have the same EHR system, developed in Sweden by Cambio Healthcare systems. These regions also provide a strong decision body for financing further developments. However, the three largest regions have not managed to co-operate but two of them, Skåne and VGR have procured the American Millenium system from Cerner but after 2 years of implementation efforts there are many issues and some uncertainty as to the eventual success of this.

The national cooperation organization SKR has created a subsidiary called Inera that is responsible for a number of services to support the regions with their ICT developments and to some extent to provide interoperability. The provision of Patient Access to their Electronic Health records is in Sweden largely managed through a common patient portal managed by Inera where patients are authenticated and are given access to the records provided by the different healthcare providers, mainly the 21 regions.

The decision making of the national Inera company is based on trying to obtain consensus and financing by the regions which has not always been easy. In general, the ICT budgets of the regions are more than ten times that of Inera.

In one area regarding management of electronic prescriptions for medicinal products, another body has an important role, the eHealth Authority which is a government agency ruled by the ministry of health and welfare. This body keeps a prescription database and connects all pharmacies allowing prescriptions to be filled at any pharmacy of the country.

# Country: Finland

## Government structure

Parliamentary democracy

## Internationally

Finland is a member of the European union and OECD[[2]](#footnote-3).

## Healthcare system

The Finnish healthcare system is based on public healthcare services to which everyone residing in the country is entitled. According to the Constitution of Finland, the public authorities shall guarantee for everyone adequate social, health and medical services. Until 2023, healthcare in Finland consisted of a highly decentralized three-level publicly funded healthcare system and a smaller private sector providing occupational healthcare and selected elective hospital services. Until 2023 around 300 municipalities were responsible for providing primary healthcare to their residents. Typically they provided primary care services themselves and specialized care via joint municipal authority owned hospitals. This system has, however, changed in the social and healthcare reform. After the reform, there are 21 new wellbeing services counties in Finland and the City of Helsinki. These organizations will organize health, social and rescue services for citizens in their area. The wellbeing services counties have assumed responsibility for organising services and started operating on 1 January 2023. The population sizes of counties vary from 68 000 to 630 000.

## Primary care

Public primary healthcare refers to the municipally arranged services and they include monitoring of the health of the population; promoting wellbeing and health; prevention, diagnosis and treatment of diseases, in particular public health diseases. Public primary healthcare services are provided at municipal health centres. In addition, private healthcare providers provide primary services for a substantial part of the employed population.

## Steering of Health ICT developments

The national PAEHR solution in Finland is provided by the government agency Social Insurance Institution of Finland (Kela). While Omakanta is the single most broadly used PAEHR in Finland, public regional and private healthcare providers also offer their patient portals that often include some interactional features such as appointment booking or symptom assessment, not included in Omakanta. While major decisions on regional Electronic Health Systems have been made independently by municipalities and hospital districts, there are some national initiatives that have succeeded to establish digital services to the citizens: Health Village has been developed by all 5 university hospitals lead by the biggest, Helsinki Uusimaa hospital district. Omaolo is a digital service developed in collaboration with several big municipalities in Finland.

# Country: Norway

## Government structure

Norway is a constitutional monarchy with the official head, at the time being a king, having only a symbolic role. Norway has a parliamentary system, which means that the government, the executive part of the system is accountable to the Storting, the legislative part. The Storting is Norway's national assembly. It consists of 169 representatives elected from 19 constituencies. Parliamentary elections are held every four years. All Norwegian citizens who have reached, or will turn 18 during the election year have voting rights.

The Ministry of Health and Care Services takes political decisions into practice for the healthcare sector.

Norway is divided into counties and municipalities. After a large government reform up to 2020, aiming to “build larger and stronger local governments” by diminishing the number of counties and municipalities,  there are now 11 counties and 356 municipalities.

## Internationally

Norway is not a member of the European Union however, Norway is a member of EEA, allowing for goods, services, capital, and persons to move freely from EU countries. Norway is also a member of OECD and NATO.

## General healthcare system

The Norwegian healthcare system is organized into two levels. The state is responsible for the specialist service, and the municipalities are responsible for the primary care. The healthcare system is providing healthcare for all citizens, according to the goal of equal access to healthcare for all. Norway has universal health and social insurance coverage, funded by general taxes. The patients pay a low fee/copayment for most services. Around 10 % of Norwegian citizens have also bought private insurance, to gain access to healthcare quicker. Norway is divided into four health regions. Each health region is led by a regional health enterprise/authority (RHF), and includes several health enterprises (HF).  Each health enterprise includes one or several hospitals and health institutions. The specialist health service includes somatic and  mental healthcare and ambulance services. The healthcare organizations mainly offer services at public hospitals, but purchase around 10 % of the healthcare services from private institutions. Patients have electronic access to health records from public hospitals

## Primary healthcare

The municipalities are responsible for primary healthcare. In 2001 the GP scheme was introduced. The aim of the GP scheme is to improve the quality of general medicine by providing all citizens with continuity in care by offering all the citizens the right to register with their own GP. The GPs are the first line in the Norwegian healthcare system, and function as a gatekeeper to welfare goods such as sick leave and referrals to specialist care. There are approximately 5000 GPs in Norway. Norway has in recent years faced capacity problems within the GP scheme, leaving around 175 000 patients without a GP (Aug 2022). The health record from the GP is not shared with the patient electronically. Other services that the municipalities are responsible for are emergency centers providing out-of-hours services, healthcare clinics for children, rehabilitation, and other health and care services such as home care.

## Steering of Health ICT developments

In Norway, the Electronic Health Record (EHR) has been fully established for many years, and the patient is both the object and the owner of the health record. Patients have, since 2001, had the right by law to access their health record and, in 2013, a White Paper,  St. 9 (2012–2013) One citizen – one health journal, stated that patients should have digital access.

Norwegian Health Network, established in 2004, is a state enterprise, owned by the Ministry of Health and Care, where the aim is to secure the digital infrastructure and communication in the health and care sector.

In 2016 the Directorate for e-health was established. The reasoning for funding a designated directorate for e-health was to enable and facilitate a national policy on e-health. The Directorate for E-health is responsible for steering and coordinating e-health with the relevant stakeholders in the healthcare system, and to develop and implement a national policy.

In 2020, around 200 employees were transferred from the Directorate for e-health to Norwegian Health Network, together with the responsibility for being the national service provider of e-health services. This includes developing, managing and operating the national e-health solutions and infrastructure. Norwegian Health Network took over tasks related to e-prescriptions, core records, basic data and the national health portal helsenorge.no.

One of the main purposes of the White paper St. 9 was to provide “health personnel with simple and secure access to patient and user information”. This means that all health personnel should have access to all necessary health data both at the primary- og the specialist care level. The Health Platform, Helseplattformen, will introduce a common electronic patient record for the entire health service in central Norway, that is to say health organizations, municipalities, GPs and private actors. The goal is a more coherent healthcare service for patients and employees throughout the region.Through the Health Platform, Central Norway is a regional testing ground for the national target image "One citizen - one health record".

# Country: Estonia

## Government structure

Estonia is a parliamentary republic. The head of government is the prime minister, who is nominated by the president and approved by parliament. The prime minister is in charge of the executive power given to the government. The head of state is the president who is elected by Parliament (Riigikogu) or electoral college for five years. The president is a ceremonial figure who does not even have nominal executive powers. The president is obliged to suspend his or her membership in any political party for the term in office.

The Parliament has 101 seats, elected every four years. The present government is led by prime minister Kaja Kallas and composed of 3 parties– Reform, Social Democratic, and Eesti 200 parties.

The country is divided into 15 counties (Regions) and 79 municipalities. The representative body of the municipal government is the council, which is elected in free elections for four years. The council has the right, within the limits of the law and in the interests of the local residents, to decide on every issue within the competence of the local government. The local government has an independent budget and the right to impose and collect taxes. The budget of the municipality is independent, which means that the municipal and city budgets are separated from the state budget and thus being part of the public sector budget, but do not form part of the state budget. The share of Estonian local government budgets in the public sector is about 25%. Local budget expenses are mainly divided into education (>40%), economic expenses, supporting leisure, culture and religion, general management costs and social protection. Local governments finance healthcare only to a very limited extent.

## Internationally

Estonia is a member of the European Union, NATO and OECD.

## General healthcare system

In Estonia, the provision of healthcare services is regulated by the Healthcare Services Organization Act, which stipulates the requirements for the organization and provision of healthcare services and the procedure for healthcare management, financing and supervision. The medical care is divided into three levels: primary or family medical care, specialized medical care and nursing care. The Estonian healthcare system is funded through mandatory contributions made through a payroll tax. Out of pocket payment by patients forms 22%. The Estonian Health Insurance Fund (EHIF) is an independent body that acts as the sole purchaser of medical care. The health insurance system covers about 95% of the population. Contributions are proportional to employment and salaries, but non-contributing citizens represent almost half of the insured people. The Ministry of Social affairs covers uninsured persons and ambulance services.

Estonia’s health system benefits from a strict separation of functions. The main actors are the Ministry of Social Affairs and its agencies, the EHIF, and independent provider units operating under private law (so-called autonomized units). The Ministry of Social Affairs and its agencies perform the main stewardship role for the Estonian healthcare system, including the development of national healthcare policies and legislation, supervision of compliance with legal acts, collection and analysis of data on activity volumes and economic indicators of providers, as well as registration of healthcare professionals and licensing of facilities. The Ministry of Social Affairs is also responsible for financing emergency care for the uninsured, as well as ambulance services and public health programs. Both the Ministry of Social Affairs and local governments finance social care.

All healthcare providers are independent entities operating under private law. Family physicians operate as private entrepreneurs or salaried employees of private companies owned by family doctors or local municipalities. Most hospitals are either limited liability companies owned by local governments or foundations established by the state, municipalities or other public agencies. The remaining few are privately owned.

Secondary and tertiary care is provided in hospitals and outpatient centers. There are currently about 65 public and private hospitals in Estonia, including 35 nursing and rehabilitation hospitals. The EHIF has contracts with the 19 public hospitals that are included in the Hospital Network Development Plan (HNDP). HNDP hospitals are divided into regional, central, general, and local. Regional hospitals deliver the full range of services, central hospitals deliver most services with the exception of a few procedures, general hospitals provide 24/7 emergency care, intensive care and some surgical and medical specialties, while local hospitals deliver 24/7 emergency and some general surgery procedures. The EHIF also has contracts with other non-HNDP hospitals, including hospitals specialized in nursing or rehabilitation care.

The majority of ambulatory specialist care is provided in hospital outpatient departments, with the remainder provided by health centers or specialists practicing independently. Day care, which is defined as treatment requiring at least a four-hour stay without the need for the patient to stay overnight, is provided by hospitals and ambulatory care providers with a day-care license. Rehabilitation care (inpatient and outpatient) is provided by licensed hospitals.

## Primary Care

Primary care is provided in Estonia by family physicians and is at the center of the health service delivery system. Family physicians serve as the first level of contact who coordinates, manages, and authorizes all health services provided to a person covered by a universal health coverage provided through Estonian Health Insurance Fund (EHIF). There are currently about 800 family physician practices in Estonia, approximately 70% of family physicians work in solo practices. Each family physician’s practice list cannot contain more than 2000 patients or less than 1200 patients. All together, these practice lists cover the entire population. In addition, family doctors and nurses provide more than half of all ambulatory care visits, while ambulatory specialists deliver the remainder of these visits (ref: https://www.haigekassa.ee/en/people/health-care-services/estonian-health-care-system).

## Steering of Health ICT developments

Estonian digital health landscape could be divided into two main domains: 1) central databases, services, and applications, and 2) databases and applications of healthcare facilities and other healthcare stakeholders. In the 1st domain, the main components are the nationwide Health Information System (EHIS) which is a centrally governed state database regulated by the Healthcare Services Organization Act and its amendment, and the database of Estonian Health Insurance Fund. The second domain is formed by the Electronic Medical Records (EMR) and Hospital Information Systems (HIS) of healthcare facilities and different applications provided and maintained by private companies. The healthcare facilities have to send digital data and documents stipulated by the legal acts to EHIS. EMR-s and HIS-s of the facilities are provided by the different private information technology companies and are competing in the digital health market. However, they have to be compliant with the central systems and be able to exchange digital data and documents according to the legal regulations.

1. Sweden joined NATO March 7, 2024, after data collection for this study was completed. [↑](#footnote-ref-2)
2. Finland joined NATO April 4, 2023, after data collection for this study was completed. [↑](#footnote-ref-3)
